# Stabilization protects islet integrity during respirometry in the Oroboros Oxygraph-2K analyzer

**DOI:** 10.1080/19382014.2022.2054251

**Published:** 2022-03-25

**Authors:** Justin J. Crowder, Ziqian Zeng, Alissa N. Novak, Nathan J. Alves, Amelia K. Linnemann

**Affiliations:** a Department of Pediatrics, Indiana University School of Medicine, Indianapolis, IN, USA; b Department of Emergency Medicine, Indiana University School of Medicine, Indianapolis, IN, USA; c Weldon School of Biomedical Engineering, Purdue University, West Lafayette, IN, USA; d Department of Biochemistry & Molecular Biology, Indiana University School of Medicine, Indianapolis, IN, USA; e Indiana Center for Diabetes and Metabolic Diseases, Indiana University School of Medicine, Indianapolis, IN, USA

**Keywords:** Diabetes, mitochondria, respirometry, islets, 3D-printing

## Abstract

Metabolic dysfunction of β-cells has been implicated as a contributor to diabetes pathogenesis, and efforts are ongoing to optimize analytical techniques that evaluate islet metabolism. High-resolution respirometry offers sensitive measurements of the respiratory effects of metabolic substrates and customizable manipulation of electron transport chain components, though the delicate nature of islets can pose challenges to conventional analyses. An affordable and reliable option for respirometry is the Oroboros Oxygraph-2 K system, which utilizes a stir bar to circulate reagents around cells. While this technique may be suitable for individual cells or mitochondria, the continual force exerted by the stir bar can have damaging effects on islet integrity. Herein, we demonstrate the protective benefits of a novel 3D-printed islet stabilization device and highlight the destructive effects of conventional Oxygraph analysis on islet integrity. Islet containment did not inhibit cellular responses to metabolic modulatory drugs, as indicated by robust fluctuations in oxygen consumption rates. The average size of wild-type mouse islets was significantly reduced following a standard Mito Stress Test within Oxygraph chambers, with a clear disruption in islet morphology and viability. Alternatively, containment of the islets within the interior chamber of the islet stabilization device yielded preservation of both islet morphology and increased cell viability/survival after respirometry analysis. Collectively, our study introduces a new and easily accessible tool to improve conventional Oxygraph respirometry of pancreatic islets by preserving natural islet structure and function throughout metabolic analysis.

## Graphical abstract

**Figure:**
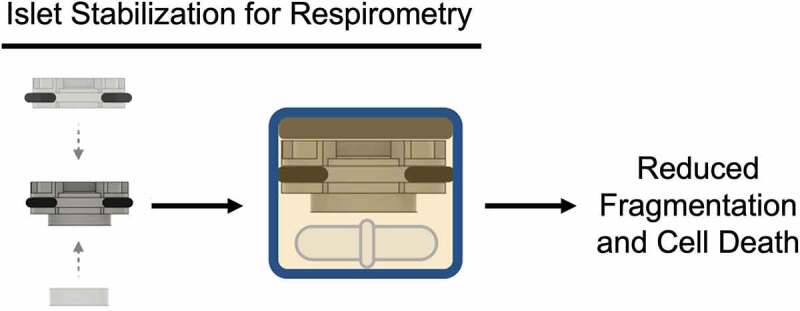

## Introduction

A hallmark of all forms of diabetes is the loss of β-cell function and/or mass. However, the initial events that trigger β-cell dysfunction are largely unknown. A number of studies suggest the involvement of impaired mitochondria during the early stages of both type 1 and type 2 diabetes.^1,2^ Therefore, analysis of islet bioenergetics and studies that address the consequences of gene mutation, diet, and environmental insults on islet mitochondrial function have the potential to provide a greater understanding about the relationship between mitochondrial dysfunction and diabetes development.

A variety of techniques are available to evaluate cellular metabolism.^3,4^ However, many of these techniques are destructive to cells and do not provide real time information. These limitations have been overcome with the development of the Seahorse (Agilent) and Oxygraph (Oroboros) instruments. Both instruments provide highly accurate measurements of mitochondrial function from isolated mitochondria, cultured cells, or tissues. Nonetheless, each platform has notable advantages and limitations. The Seahorse platform utilizes specially designed multi-well plates to measure glycolysis, via extracellular acidification, and oxygen consumption in an automated, high throughput manner. However, the Seahorse equipment is expensive and the requirement of using specialized plates and reagents adds to the cost. Although the Oxygraph platform is labor intensive and limited to only measuring oxidative phosphorylation, it may be more accessible to users when there are limited resources.

Recent studies utilizing the Seahorse and Oxygraph platforms have provided a wealth of information on metabolic characteristics of rodent and human islets.^5–10^ However, concerns have been raised regarding the delicate and dynamic nature of intact islets within metabolic analysis instruments.^11^ For example, respirometry analysis with the Oxygraph-2 K system (Oroboros) includes a stir bar that circulates buffer and reagents. This creates a forceful interaction between the stir bar and islets that may disrupt islet integrity, leading to data that is more indicative of respiration within individual cells, rather than intact islets. Islet stabilization within Oxygraph chambers may therefore provide protection to these fragile tissues, leading to similar benefits of intact islet analysis as those seen in Seahorse islet plates.

In the present study, we aimed to evaluate the effects of conventional Oxygraph-2 K analysis on the integrity and function of intact mouse islets and the potential benefits of islet stabilization within Oxygraph instruments. To test this, we designed a 3D-printed device to protect and stabilize islets. We show that stabilization of islets within the device maintains islet integrity which may improve reliability of data generated from Oxygraph studies, contributing to a deeper understanding of the metabolic environment that contributes to diabetes development.

## Results

### Islet stabilization does not inhibit metabolic drug responses

**Figure 1.**
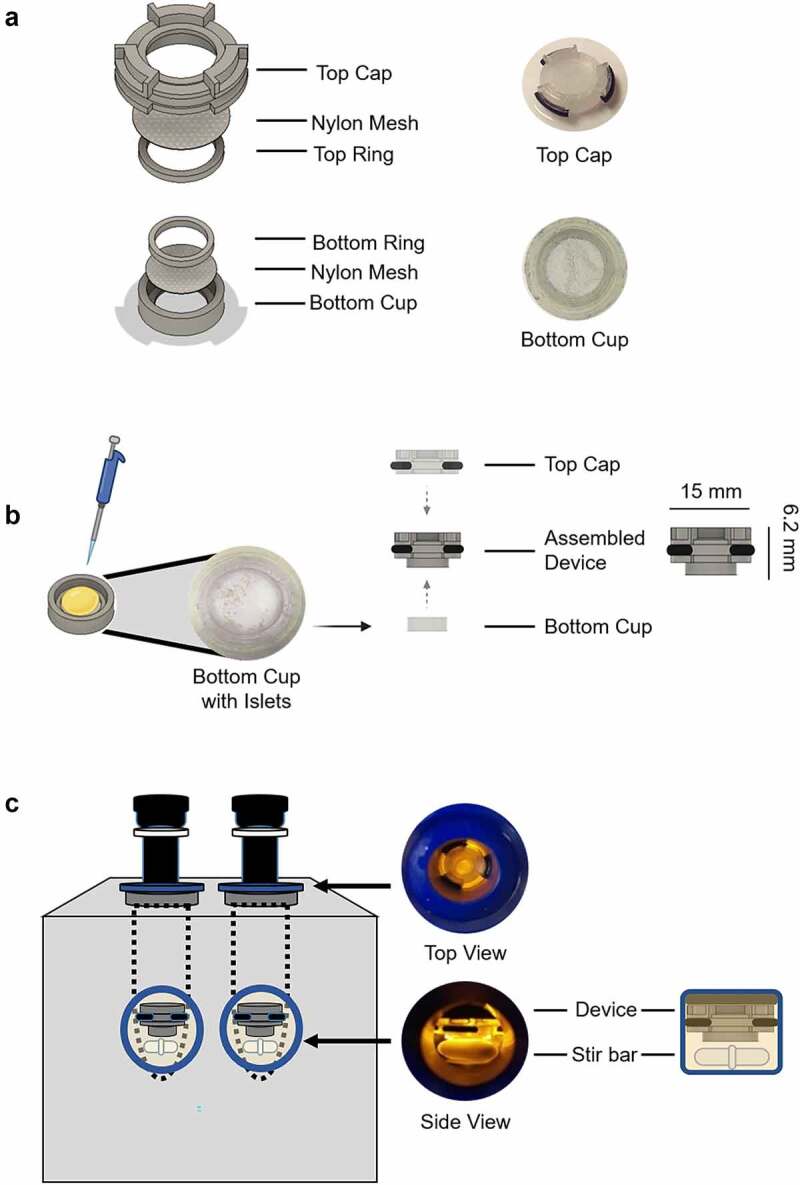
A 3D printed mesh device to stabilize islets within the Oxygraph-2 K chamber. (a) Structural components of the islet containment device are shown. The top cap and bottom cup of the device were printed on a fused deposition 3D printer (Creality3D) using transparent PLA filament. 40 µm nylon mesh was secured to both the cap and the bottom chamber using plastic rings. (b) Wild-type mouse islets were gently pipetted onto the bottom chamber and evaluated on a dissecting microscope (left). Three O-ring rubber sections were fit to the grooves of the cap, and the cap was pressure fitted onto the bottom cup containing the islets (right). (c) The entire device containing islets was loaded into an Oxygraph-2 K chamber (Oroboros) and gently submerged in Mir05 buffer to rest just above the stir bar.

To stabilize islets, a 3D-printed mesh device was designed to fit securely within the glass chambers of the Oxygraph-2 K respirometer (Figure 1(a)). Following islet isolation as previously described,^12^ 400–500 wild-type mouse islets were gently pipetted onto the bottom cup of the 3D printed device (Figure 1(b)) and evaluated for morphological integrity. The device was closed by pressure fitting a top cap onto the bottom cup. The device was then gently inserted into an Oxygraph chamber (Figure 1(c)) until it rested just above the stir bar. A comparable group of wild-type islets were directly pipetted into the neighboring Oxygraph chamber as a control and both islet groups were assessed in parallel.

**Figure 2.**
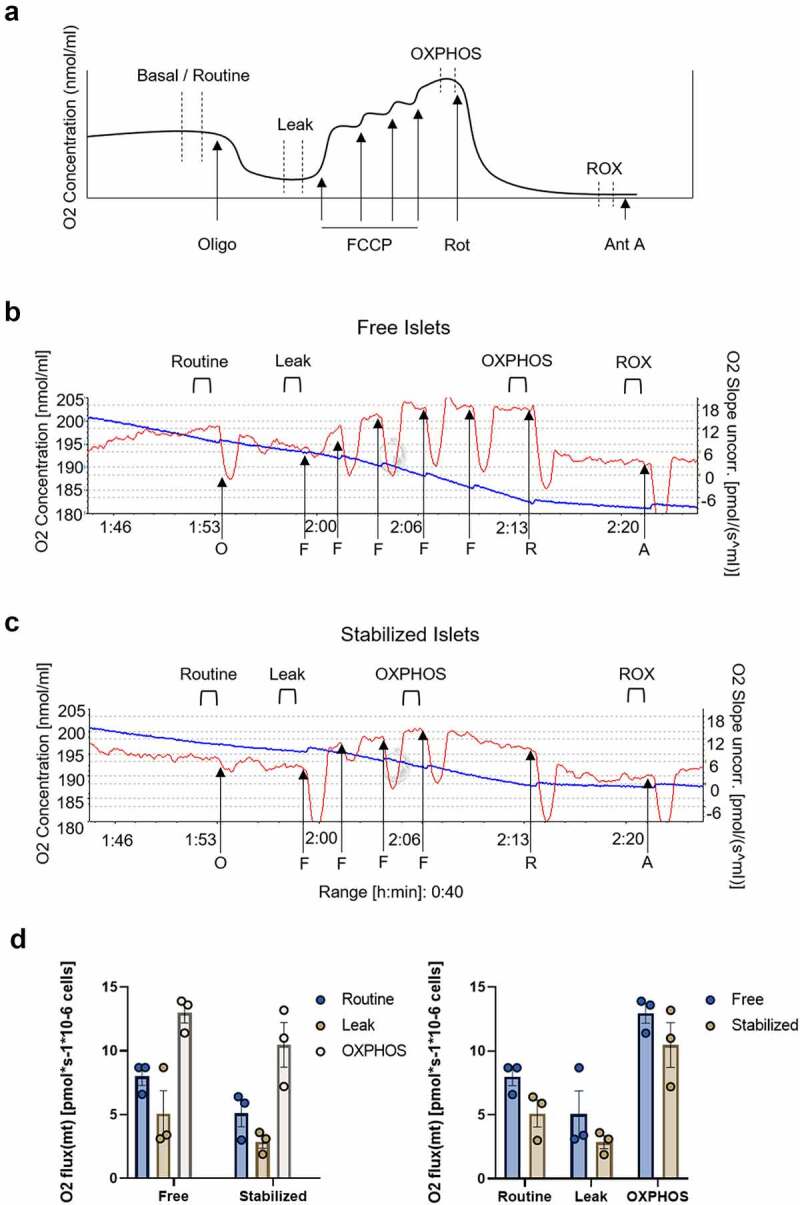
Islet stabilization does not inhibit cellular response to metabolic modulators. (a) Depiction of a standard Oxygraph respirometry trace. Anticipated oxygen fluctuation (curved line) in response to common drug injections (arrows) are shown. Areas of curve stabilization (dashed lines) are used to calculate respiratory parameters from respirometry data. (b) Representative respirometry trace from wild-type mouse islets free in the Oxygraph-2 K chamber containing Mir05 buffer. (c) Representative respirometry trace from islets in a mesh device submerged in Mir05 buffer within the Oxygraph-2 K chamber. Oxygen concentration (blue) and oxygen flux (red) are shown for each experimental trace. Metabolic drugs were sequentially added to each chamber (arrows) to evaluate parameters of mitochondrial respiration; O, Oligomycin; F, FCCP; R, rotenone; A, antimycin A. (d) Representative areas of each oxygen flux trace were selected to calculate basal respiratory rate (Routine), “leak” state, maximal respiratory capacity (OXPHOS), and non-mitochondrial respiration (ROX). Averaged oxygen flux values were normalized to ROX to calculate oxygen flux ratios.

To assess the respiratory capacity and corresponding responses to metabolic drugs, islets (400–500 per group) were subjected to a standard Mito Stress Test (Figure 2(a)) and respirometry was performed on free-floating (loose in the Oxygraph chamber; Figure 2(b)) and stabilized groups (contained in the device, Figure 2(c)) simultaneously in adjacent chambers within the same instrument. Mito Stress Tests included treatment with oligomycin (O; 2.5 µM), followed by FCCP (F; 0.05 µM), rotenone (R; 0.5 µM), and antimycin A (A; 2.5 µM). Basal oxygen consumption (Routine), proton “leak,” maximal respiration (OXPHOS), and non-mitochondrial respiratory rates (ROX) were identified for free and stabilized islets (Figure 2(d)). For both islet groups, average oxygen consumption rates (OCR) fluctuated as anticipated in response to drug treatments, with OCR values decreasing below basal state in response to oligomycin and incrementally rising above basal state following FCCP titration. While OCR values were consistently lower in stabilized islet groups, no significant difference was detected between the two groups for any metabolic parameter. These data suggest comparable respiratory capacities between free and stabilized islets, with both groups exhibiting robust responses to metabolic modulatory drugs.

### Use of a 3D-printed mesh device reduces islet fragmentation within Oxygraph-2 K chambers

**Figure 3.**
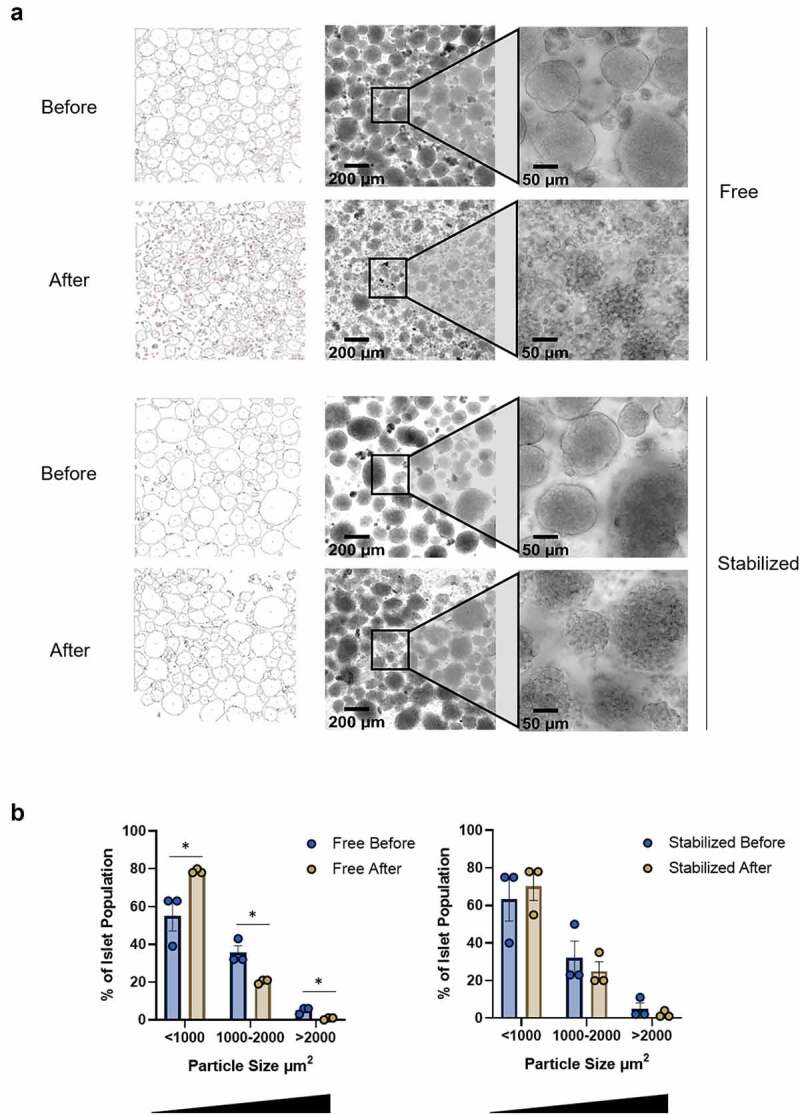
Islet stabilization reduces fragmentation. (a) Representative images of wild-type mouse islets imaged before and after free and stabilized respirometry in an Oroboros Oxygraph-2 K system. 10x (middle) and 40x (right) images were collected on a DMI8 Widefield Epifluorescence Microscope (Leica). Particle analysis (left) of free and stabilized islet populations was performed using Image J software. 10X images chosen for analysis represent approximately 11% (before) and 17% (after) of total islet populations. (B) Quantification of particle size in free and stabilized islets both before and after respirometry analysis. Data are mean ± SEM for 3 individual experiments. *P < .05; unpaired t test.

Following respirometry, free islets and stabilized islets were retrieved and evaluated under a microscope to assess islet integrity. Brightfield microscopy images reveal a decrease in fragmentation and a retention of initial islet size when islets are stabilized within the device. Free islet images collected before and after Oxygraph respirometry (Figure 3(a)) exhibit a clear disruption of islet morphology and a reduction of visibly intact islets, indicative of islet damage during the respirometry process. Statistical analysis of free islets (Figure 3(b)) revealed a 24% increase (p = .04) in smaller particles (<1,000 µm^2^) and a 20% decrease (p = .01) in larger particles (>1,000 µm^2^) following respirometry. In contrast, although the islet capsule integrity of stabilized islets is somewhat decreased following respirometry (Figure 3(a)), the abundance of large islets is maintained, and no significant change in islet size is detected via particle analysis (Figure 3(b)). Collectively, these data provide evidence of damaging interactions between islets and stir bars in Oxygraph chambers, which can be attenuated by stabilization of islets within the containment device.

### Islet stabilization promotes cell survival within Oxygraph-2 K chambers

**Figure 4.**
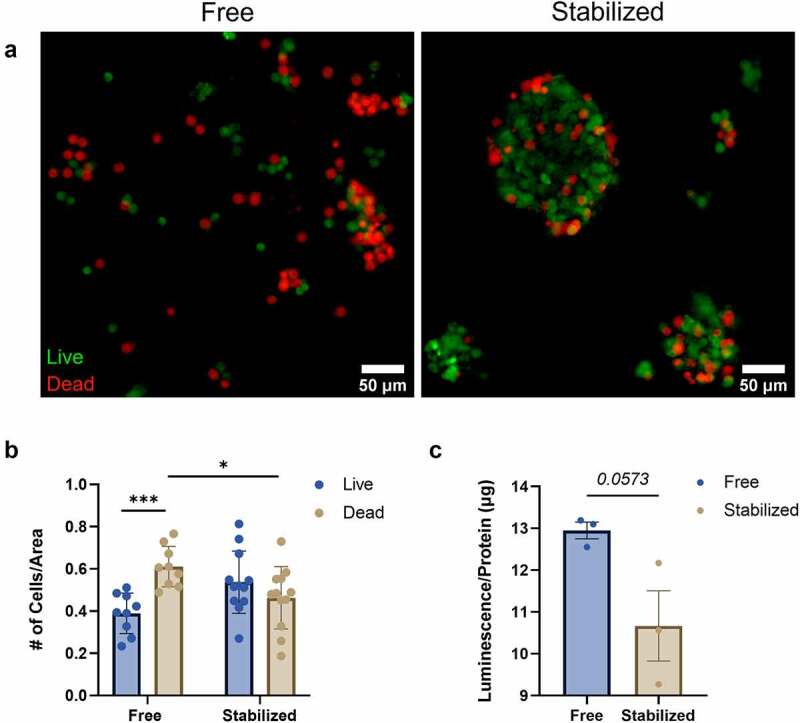
Islet stabilization reduces islet cell death during respirometry. (a) Representative images of free or stabilized islets stained with acridine Orange (AO, green, Live cells) and propidium iodide (PI, red, Dead cells) acquired using a 20X objective on a DMI8 Widefield Epifluorescence Microscope (Leica). (b) For free and stabilized islets, the number of AO/Live and PI/Dead positive cells were quantified and normalized to total area using ImageJ. (c) Caspase 3/7 activity was measured using Caspase-Glo ® 3/7 assay. Luminescent signals were collected and normalized to total protein. Data shown are mean ± SEM for multiple islets/groups of cells from 3 individual experiments. *P < .05, ***P < .001; unpaired t test.

To assess the potential benefits of islet stabilization on cell survival, cell viability assays were performed on free and stabilized islet groups. Following respirometry, islets were stained with acridine orange (AO) and propidium iodide (PI). AO is a cell permeable dye that fluoresces green upon entry into viable cells and integrates into dsDNA, whereas PI is not cell permeable and only becomes fluorescent when membrane integrity is compromised. Analysis of islets/cells stained with both AO and PI revealed a significantly greater proportion of dead (red) cells in the free islets in comparison to the device-stabilized islets (Figure 4(a,b)).

To further assess apoptosis in islets after respirometry, endogenous caspase 3/7 activity was measured using a luminescence-based assay. Caspase 3/7 activity was modestly reduced in the stabilized islet group compared to free islets (Figure 4(c)). Together, these results further highlight the destructive effects of Oxygraph stir bars on islet cell health and viability, and demonstrate the protective effects of islet containment on islet cell survival.

## Discussion

Islet-derived cells have been common sources for oximetry analyses in investigations of diabetic conditions, but evaluation of intact islets via high resolution respirometry is an emerging technique.^13^ While specially designed plates have optimized the metabolic analysis of whole islets in Seahorse instruments, efforts are still underway to validate assay parameters for islet metabolism. Current studies have used 1,000 islets or more per experimental group, and even the most precise techniques require 30–150 islets per sample. As islets are delicate and precious resources from pancreatic tissue (comprising only 1–2% of the organ), researchers with limited means may greatly benefit from techniques that minimize sample size and increase precision and reliability of metabolic measurements.

Islets are fragile organoids and preserving structure and function from isolation to downstream analyses can be challenging. This study highlights the delicate nature of islet integrity, and we propose a strategy by which islet morphology may be conserved throughout Oxygraph respirometry analysis. An additional obstacle exists in drug treatment of these 3D structures, as chemicals do not permeate islet cells in a uniform fashion and circulation of buffer is accordingly necessary. The Oxygraph-2 K instrument has addressed this concern through incorporation of a stir bar in the base of the glass chamber. However, as we show, the destructive forces associated with contact between intact islets and the magnetic stir bar can lead to islet fragmentation and cell death. The mesh device described above sequesters islets away from the destructive forces of the stir bar while maintaining circulation of reagents through the mesh and around the islets. The corresponding preservation of islet morphology and viability may provide more reliable and reproducible measurements of islet mitochondrial function for future studies utilizing Oxygraph equipment.

An inherent caveat of the Oxygraph-2 K system is the need for large sample sizes. This investigation used groups of 400–500 islets per condition, which is consistent with sample sizes from prior studies ranging from 150 to 1,000 islets per group.^6,14,15^ These large numbers are a stark contrast to Seahorse islet studies where groups of 30–70 islets are often used for 24-well islet capture plates.^10,16^ Oroboros Instruments does offer an alternative Oxygraph chamber (O2k-chamber sV, available in the O2k-sv Module) which reduces the chamber volume from 2.0 ml to 0.5 mL, thereby reducing the sample size needed for respirometry. This may be an attractive option for islet studies utilizing the Oxygraph-2 K, and its use may enable reduced islet input for reliable measurements. However, the stabilization device described herein would require resizing to allow for use in the O2k-chamber sV, and the number of islets required may still exceed that of a Seahorse islet plate. These limitations of Oroboros respirometry should be carefully considered before incorporation of the technique into islet investigations. Nonetheless, substantially reduced cost of materials and reagents and real-time customization make Oxygraph respirometry a popular choice for labs with limited resources or those looking to explore novel mechanisms of action in islets.

It may be noted that stabilized islet data suggests a trend toward decreased OCR values, perhaps indicative of a dampened drug response. Efficiency of drug permeation is a common challenge in islet metabolic studies, as indicated by frequent incorporation of permeabilization agents such as saponin and digitonin.^5,6^ Drug delivery to all cells within intact islets is a slower and more challenging process than circulation and delivery to individual cells, and thus decreased OCR values may be indicative of the maintenance of relatively normal islet integrity in stabilized islets. Thus, it should be noted that artificially forcing drug penetration using permeabilization agents may produce data that is not representative of *in vivo* function even beyond the use of isolated islets. However, although islet stabilization does not inhibit the cellular response to metabolic drugs, studies aiming to increase drug responsiveness may still benefit from application of a permeabilization agent prior to Oxygraph analysis.

A limitation of this study is that we are unable to address the potential contribution of fractionated cells to the OCR values collected in free islet experiments. Islets disrupted by Oxygraph stir bars may disperse into individual islet cells that are still capable of glucose oxidation during respirometry. We observe a considerable population of fractionated, viable cells present within free islet populations following Oxygraph respirometry. While these isolated cells may contribute to the overall OCR values of an islet experiment and could account for the decreased OCR trends seen in stabilized islets, the resulting data may not clearly reflect *in vivo* cellular function within the intact islet. This additional variable that could impact data reliability further supports the need for a mechanism of islet protection in Oxygraph experiments.

It should be emphasized that any manipulation of isolated islets may be destructive to both morphology and function, and stabilization of islets does not entirely preserve islet architecture. While the stabilization of islets yields a significant reduction in fragmentation and dead cells during the respirometry analysis, native islet morphology is still somewhat disturbed and a small number of nonviable cells can be found within intact islets, especially around the islet exterior. Several factors may contribute to this morphological disturbance, including transport of islets into the device and damaging effects of the drugs on islet cell health. This is further evidence of the fragile nature of islets, and therefore, procedures should be identified to minimize the stress imposed on islet cells during isolation, processing, and when utilizing a containment device.

In summary, in this study we highlight the destructive effects of conventional islet respirometry using the Oxygraph-2 K instrument and present a novel device that can be used to maintain islet morphology. Although the mesh device described here was designed for islet studies, it is likely to be applicable to study any fragile tissue or cell type for respirometry. Utilizing this same stabilization device for larger tissue fragments could enable analysis in the oxygraph instrument free from the damaging effects of the stir bar and provide a unique capability of this approach by expands utility of stabilization beyond just isolated islets. Our data suggest that utilization of a stabilization device may increase reliability of metabolic studies while conserving valuable resources. Thus, the modified islet respirometry techniques presented here may provide a valuable and widely accessible approach to investigation of metabolic dysfunction in diabetes pathogenesis.

## Materials

MgCl_2_, KH_2_PO_4_, and HEPES were purchased from Fisher Scientific (Waltham, MA). 40 µm nylon mesh was purchased from Gilson Company (Lewis Center, OH). A 1.75 mm polylactic acid (PLA) filament was purchased from Creality3D (Shenzhen, China). All other materials were purchased from Sigma-Aldrich Chemicals (St. Louis, MO) unless otherwise specified.

### Methods

#### Mice

All experiments in this study were approved by the Indiana University School of Medicine Institutional Animal Care and Use Committee. C57BL/6J wild-type mice were housed in a temperature-controlled facility with a 12 hr light/12 hr dark cycle and were allowed free access to food and water. Male and female mice between the ages of 10 to 20 weeks were selected for analysis.

#### Islet isolation

Mice were sacrificed via cervical dislocation, and pancreatic islets were isolated by collagenase digestion as previously described.^12^ In brief, pancreata were inflated via injection of collagenase XI into the common bile duct, and islets were separated from exocrine tissue. Islets were then handpicked under a dissecting microscope and cultured overnight at 37°C for subsequent analysis.

#### Production of mesh device

The reusable mesh device was designed in an open-source CAD software and printed using transparent PLA filament through a fused deposition 3D printer (Ender 3 Pro, Creality3D). Due to the small size of the parts, printing speed was set at 5 mm/s. The mesh device was assembled through pressure fitting four 3D printed components, including a supporting cap (3 mm x 15 mm), a bottom cup (2.5 mm x 9.7 mm), and two plastic rings (9.7 mm top, 7.4 mm bottom) to secure two 40 µm nylon meshes (9.5 mm top, 7.9 mm bottom) on each side of the chamber. Three small rubber O-ring sections (1 mm) were fit to the grooves of the cap to hold the device in position when being placed into the Oroboros chamber. The device was specifically designed to allow fluid flow. Since fluid can flow through the mesh as well as around the device, the stir bar below the device is capable of rapidly mixing added reagents without disturbing the islets contained between the mesh components.

#### High-resolution islet respirometry

Islets from 3 to 4 mice (400–500 islets total) were centrifuged for 5 minutes at *300 x g* and pooled into a single 35 mm dish for imaging prior to respirometry analysis. After pre-respirometry imaging, islets were pipetted either directly into MiR05 buffer (3 mM MgCl_2_, 60 mM lactobionic acid, 20 mM taurine, 10 mM KH_2_PO_4_, 20 mM HEPES, 110 mM Sucrose, 0.1% BSA, 5.5 mM glucose, 0.05 mM octonoate) within a high-resolution oxygraph chamber (Oxygraph-2 K, Oroboros Instruments, Innsbruck, Austria) or into the bottom cup of the mesh device. The presence and integrity of stabilized (device) islets were evaluated on a dissecting microscope prior to addition of the device cap, and the device was submerged in MiR05 buffer in the second oxygraph chamber. Free and stabilized islets were analyzed simultaneously in the neighboring chambers, and magnetic stirring was begun once islets were secured and chambers were closed. All calibrations and experiments were conducted at 37°C. Oxygraph islet respirometry was conducted as depicted in Figure 2(a). Oxygen concentration and oxygen flux were recorded using DatLab software (Oroboros). Baseline oxygen consumption rates were identified for each chamber, after which oligomycin (2.5 µM) was added to inhibit ATP synthase and assess uncoupled respiration (“Leak” state). The protonophore carbonyl cyanide p-(trifluoromethoxy) phenylhydrazone (FCCP) was then titrated in 0.05 µM (1 µl) increments until peak oxygen flux was achieved, indicative of maximal islet respiration. Finally, 1 µl each of rotenone (0.5 µM) and antimycin A (2.5 µM) were sequentially added to inhibit ETC complexes I and III, respectively, and identify non-mitochondrial islet respiration. Following respirometry, islets were either pipetted out of the oxygraph chamber or flushed from the device with islet media then collected for subsequent analysis.

#### Cell viability assays

Following respirometry, islet groups were centrifuged at *300 x g* for 5 minutes and resuspended in 400 µl of MiR05 buffer. 100 µl was removed for acridine orange/propidium iodide (AO/PI) analysis. AO/PI was added to samples in a 35 mm glass bottom dish (MatTek Corporation, Ashland, MA) and incubated for 15 minutes in the dark prior to imaging on a Lecia DMI8 Widefield Epifluorescence microscope The remaining 300 µl of islet suspension was washed in 1X PBS then resuspended in 300 µl 1X PBS. 50 µl aliquots were added in triplicate to a white flat bottom 96-well dish (Corning, Corning, NY). 50 μl of Caspase-Glo 3/7® assay reagent (Promega, Madison, WI) was then added to each well and incubated for 1 hour in the dark. Luminescence was measured on a SpectraMax M5 microplate reader (Molecular Devices, San Jose, CA). Luminescence data were normalized to total protein content. Corresponding graphical data were generated and analyzed using GraphPad Prism v.9.1.

#### Microscopy and image analysis

Transmitted images of islets were collected using 10x (HC PL FL 10x/0.32 PH1), 20x (HC PL FL L 20X/0.40 PH1), and 40x (HC PL FL L 40x/0.60 PH2) objectives on a Lecia DMI8 Widefield Epifluorescence microscope equipped with a DFC9000 GT camera (Leica Microsystems Inc., Buffalo Grove, IL) before and after respirometry to evaluate islet morphology, integrity, and viability. 10X mosaic images of whole islet populations were collected before and after respirometry, and mosaic islet area was used to determine the percent area of representative 10X images. Images were obtained and analyzed with LAS X software. Image processing was performed on representative 10x and 20x images using Image J software.^17^ Corresponding graphical data were generated and analyzed using GraphPad Prism v.9.1.
